# Diagnostic Accuracy of Transcranial Sonography of the Substantia Nigra in Parkinson’s disease: A Systematic Review and Meta-analysis

**DOI:** 10.1038/srep20863

**Published:** 2016-02-16

**Authors:** Dun-Hui Li, Ya-Chao He, Jun Liu, Sheng-Di Chen

**Affiliations:** 1 Department of Neurology & Institute of Neurology, Ruijin Hospital affiliated to Shanghai Jiao Tong University School of Medicine, Shanghai, 200025 China

## Abstract

A large number of articles have reported substantia nigra hyperechogenicity in Parkinson’s disease (PD) and have assessed the diagnostic accuracy of transcranial sonography (TCS); however, the conclusions are discrepant. Consequently, this systematic review and meta-analysis aims to consolidate the available observational studies and provide a comprehensive evaluation of the clinical utility of TCS in PD. Totally, 31 studies containing 4,386 participants from 13 countries were included. A random effects model was utilized to pool the effect sizes. Meta-regression and sensitivity analysis were performed to explore potential heterogeneity. Overall diagnostic accuracy of TCS in differentiating PD from normal controls was quite high, with a pooled sensitivity of 0.83 (95% CI: 0.81–0.85) and a pooled specificity of 0.87 (95% CI: 0.85–0.88). The positive likelihood ratio, the negative likelihood ratio and diagnostic odds ratio were calculated 6.94 (95% CI: 5.09–9.48), 0.19 (95% CI: 0.16–0.23), and 42.89 (95% CI: 30.03–61.25) respectively. Our systematic review of the literature and meta-analysis suggest that TCS has high diagnostic accuracy in the diagnosis of PD when compared to healthy control.

Parkinson’s disease (PD) is the second most common neurodegenerative disease and is clinically characterized by resting tremor, rigidity, bradykinesia, and abnormal gait and posture. The gold standard for the diagnosis of PD is post-mortem neuropathological examination, which unfortunately precludes impactful clinical decision making to alleviate a PD patient’s symptoms^1^. Consequently, the diagnosis of PD is mostly based on clinical manifestations and expertise, which results in a large cohort of PD patients unidentified^2^. Therefore, a reliable and convenient test that recapitulates the clinical diagnosis of PD and identifies subclinical PD patients is needed in order to facilitate early disease management and delay or prevent the progression of PD.

Ultrasonography has been well-established as a diagnostic method in general medicine for over five decades. However, ultrasonography had not been applied to movement disorders due to the impenetrability of intact skull bones, until Becker first reported a specific high echogenic area within the substantia nigra (SN) in PD patients^3^. Since then, numerous studies have focused on the echogenicity of the SN and the diagnostic accuracy of transcranial sonography (TCS) in distinguishing PD patients from healthy controls, or other movement disorders. Nevertheless, the sensitivity and specificity of TCS in PD varied widely due to racial differences, sample size and diverse ultrasound devices. In a cross-sectional study conducted in Italy, using a 2–4 MHz probe, researchers found the sensitivity and specificity of TCS in diagnosing PD to be 62.71% and 76.92%, respectively^4^, while the value reported by Maria Sierria *et al.* was 95.50% and 84.78%, respectively^5^. Unfortunately, the lack of a comprehensive evaluation of the clinical utility of TCS has prevented the application of this non-invasive, non-radioactive and convenient technique in routine clinical practice. Therefore, the purpose of the present study is to perform a systematic literature review and meta-analysis to assess the overall diagnostic accuracy of TCS in the diagnosis of PD.

## Methods

### Search strategy

A systematic and comprehensive literature search using Pubmed, ISI Web of Science, EMBASE, Cochrane Library databases, and CNKI (a Chinese database), from 1966 until March 2015, was conducted for all the existing literatures regarding the diagnostic accuracy of TCS in the diagnosis of PD. The Medical Subjective Heading (MeSH) terms or keywords “transcranial sonography” and “Parkinson’s disease” were used. Subsequently, only studies published in English or Chinese were evaluated. Repeat articles were manually deleted. If an article did not present complete data, a request for raw data was sent to the original authors via e-mail. In addition, an earnest attempt to acquire unpublished data was made but no studies were appropriate for inclusion. This work was performed by two independent authors (Li and He).

### Eligibility and Exclusion criteria

Two authors carefully read and evaluated all of the articles independently. Studies were included in the current review if they met the following criteria: 1) Cross-sectional study that evaluated the ability of TCS of the SN to distinguish PD patients from healthy controls; 2) Cross-sectional study that compared SN echogenicity between patients with PD, essential tremor, or other movement disorders. Review articles, conference reports, letters, editorial comments, opinions, preface, and articles not published in English or Chinese were excluded. Other exclusion criteria for the current systematic review were: 1) articles focused on therapy and management of PD; 2) articles on Parkinsonism or other diseases, but not idiopathic PD; 3) studies that did not contain a healthy control group; 4) studies investigating the pathogenesis of SN echogenicity; 5) epidemiological studies of TCS in community dwelling elders. Two independent investigators evaluated the eligibility of all included studies.

### Data extraction, Quality assessment and Statistical analysis

All relevant data of the 31 studies, including: the first author, the year when the study was carried out, diagnostic criteria of PD, ultrasound device, number of true positives, false negatives, true negatives, and false positives were extracted in a unified form. Any divergence in this procedure was resolved by discussion. The revised version of the Quality Assessment of studies of Diagnostic Accuracy Studies (QUADAS-2), with 4 key domains containing 11 items^6^, was used to assess the quality of all included studies. Each domain facilitates assessment of the risk of bias and applicability of the primary investigation. Two authors performed the quality assessment independently, with disagreements resolved by discussion or appealing to a third author.

The statistical software Meta-Disc, version 1.4 for windows (XI Cochrane Colloquium, Barcelona, Spain) and STATA, version 12.0 (Stata Corporation, College Station, TX, USA) were used in the present study. To explore potential heterogeneity arising from the threshold effect, we computed Spearman correlation coefficients between sensitivity and 1-specificity. For any possible non-threshold heterogeneity, we applied the chi-square-based Q test and the inconsistency index *I*^2^. A significant Q test (*I*^2^ value > 50%) identifies a moderate or high degree of heterogeneity^7^. Subsequently, a random-effect model (DerSimonian Laird method) was used to calculate the pooled sensitivity, specificity, diagnostic odds ratio (DOR), and other related indexes. Otherwise, the Mantel-Haenszel fixed effect model was utilized. In order to assess the source of heterogeneity, we used subgroup analysis according to different threshold variables when heterogeneity arose from the threshold effect, and sensitivity analysis was chosen for non-threshold heterogeneity. Furthermore, meta-regression was implemented to investigate the source of heterogeneity within the included studies. We produced Deeks’ funnel plot to test the potential publication bias in our study, with a *p* value < 0.1 suggesting significance^8^.

## Results

### Characteristics and quality of the included studies

**Figure 1:**
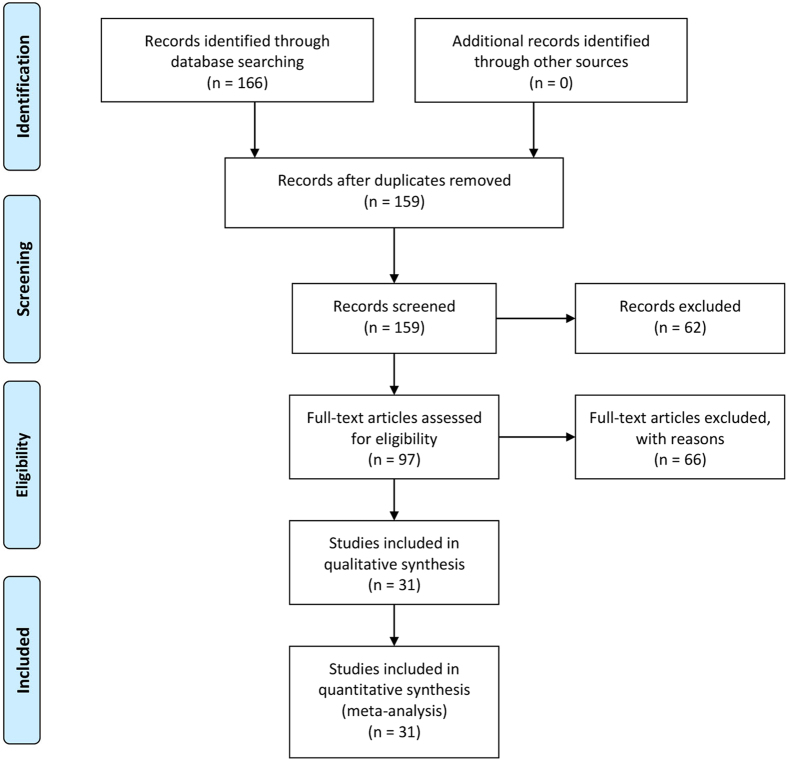
Flow chart of the selection process of included studies.

The inclusion and exclusion criteria for article selection are illustrated in Fig. 1. Ultimately, 31 studies^4^,^5^,^9^,^10^,^11^,^12^,^13^,^14^,^15^,^16^,^17^,^18^,^19^,^20^,^21^,^22^,^23^,^24^,^25^,^26^,^27^,^28^,^29^,^30^,^31^,^32^,^33^,^34^,^35^,^36^,^37^ containing 1,926 idiopathic PD patients and 2,460 healthy controls from 13 countries, were included in our meta-analysis. The main characteristics of the included studies are summarized in Table 1.

### Diagnostic accuracy

**Figure 2:**
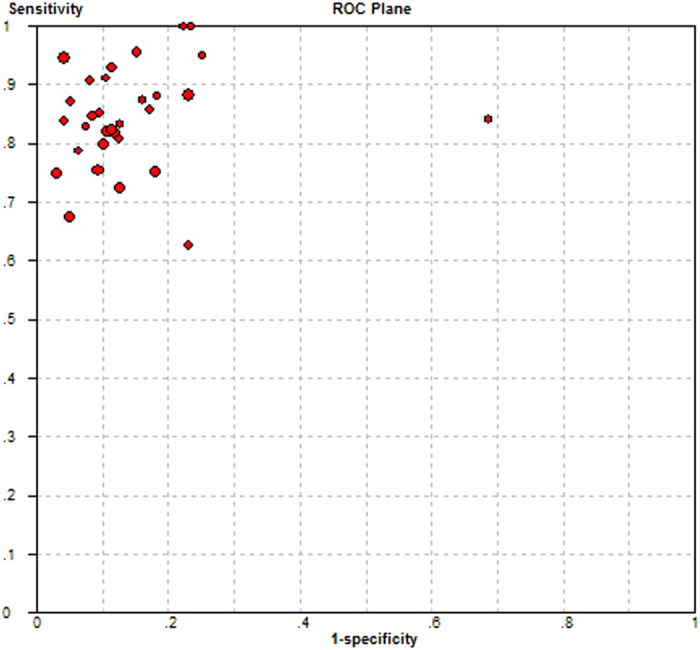
Sensitivity versus 1-specificity in receiver operating characteristic (ROC) plane for each eligible study.

**Figure 3:**
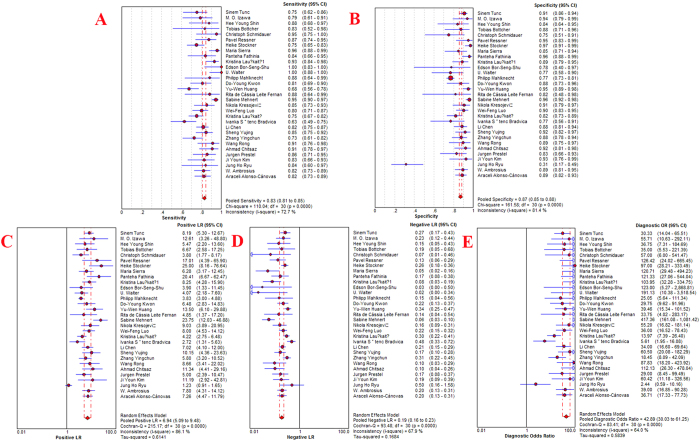
Forest plots of the diagnostic accuracy of the transcranial sonongraphy of the substantia nigra in Parkinson’s diseases. A = Sensitivity; B = Specificity; C = Positive LR; D = Negative LR; E = Diagnostic OR. CI = confidence interval; LR = likelihood ratio; OR = odds ratio.

**Figure 4:**
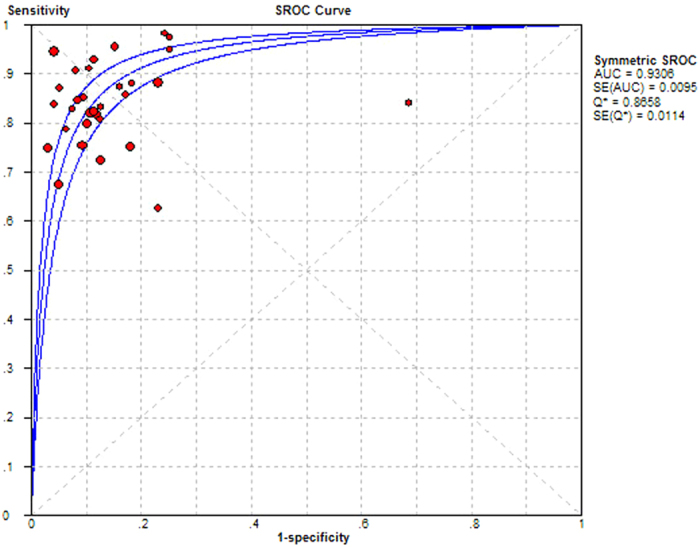
Summary receiver operating characteristic (SROC) curve for transcranial sonography of the substantia nigra in the diagnosis of Parkinson’s disease for all studies. AUC = area under curve; SE = standard error; Q* = point at which sensitivity and specificity are equal.

Statistical analysis revealed no heterogeneity secondary to the threshold effect, as the ROC plane did not have the typical “shoulder arm” pattern (Fig. 2) and the Spearman correlation coefficient of sensitivity and 1-specificity was 0.289 (*p* = 0.115). However, there was significant heterogeneity across the studies in sensitivity, specificity, positive likelihood ratio (PLR), negative likelihood ratio (NLR) and Diagnostic Odds Ratio (DOR), with an *I*^2^ index of 72.7% (*p* < 0.0001), 81.4% (*p* < 0.0001), 86.1% (*p* < 0.0001), 67.9% (*p* < 0.0001) and 64.0% (*p* < 0.0001), respectively. Overall, the diagnostic accuracy of TCS for the diagnosis of PD among patients versus healthy controls was measured based on the pooled sensitivity of 0.83 (95% CI: 0.81–0.85), pooled specificity of 0.87 (95% CI: 0.85–0.88), pooled PLR of 6.94 (95% CI: 5.09–9.48), pooled NLR of 0.19 (95% CI: 0.16–0.23) and pooled DOR of 42.89 (95% CI: 30.03–61.25) using the random effects model. The forest plots of all the indices are displayed in Fig. 3. The overall high level of accuracy is reflected by the symmetric SROC curve with an AUC of 0.9306 (standard error: 0.0095) and Q-value of 0.8658 (standard error: 0.0114) (Fig. 4).

### Meta-regression analysis

Meta-regression analysis was utilized to investigate potential reasons for inter-study heterogeneity based on geographical location (Europe, Asia or America), sample size (<50 or ≥ 50), age of PD patients (<65 or ≥ 65), ultrasound equipment (<2.5 MHz or ≥ 2.5 MHz), and QUADAS-2 scores (<10 or ≥ 10). However, none of the above covariates were found to be significant sources of heterogeneity, as all *p* values were > 0.05.

### Sensitivity analyses

Sensitivity analyses were performed to explore the possible heterogeneity and verify the consistency of the results from our meta-analysis by applying the leave-one-out method in which the first of the *K* studies is left out on repeat meta-analysis of the resulting subgroup containing *K*−1 studies. This analysis is repeated for the next *K* studies until all distinct meta-analyses are performed, each leaving out one study. Overall, no substantial alterations of the results were found in our investigation, with the pooled sensitivity ranging from 0.82 (95% CI: 0.80–0.84) with omission of the study by Maria Sierra 2013^5^ to 0.84 (95% CI: 0.82–0.85) with omission of the study by Yu-Wen 2007^22^, and the pooled specificity ranging from 0.86 (95% CI: 0.85–0.88) by removing the study by SinemTunc 2015^9^ to 0.89 (95% CI: 0.88–0.90) by removing the study by Philipp Mahlknecht 2013^20^. These sensitivity analyses indicate statistically consistent results with a high level of overall accuracy using TCS in the diagnosis of PD. Moreover, among the included studies, no single study was found to be the source of heterogeneity.

### Evaluation of publication bias

**Figure 5:**
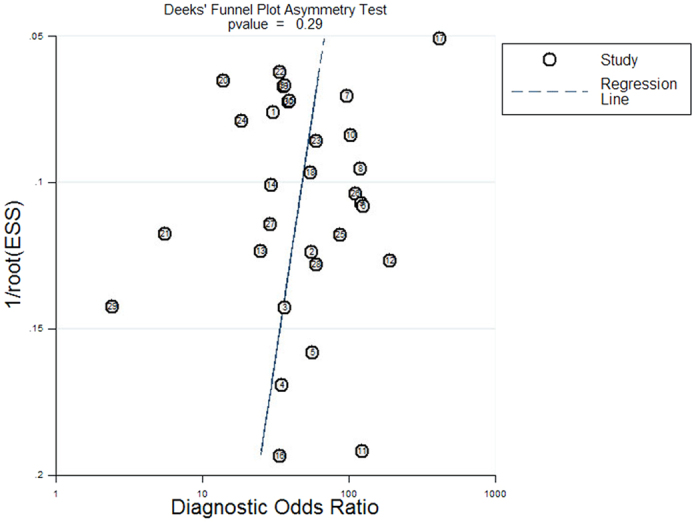
Funnel plot for the assessment of the potential publication bias of the 31 included studies. Each solid circle represents each study in the meta-analysis. The line indicates the regression line.

Deeks’ funnel plots were produced to explore the potential presence of publication bias. Based on the symmetric shape of the funnel plot of pooled DOR (Fig. 5) and the Deeks’ test non-significant value (*p* = 0.29), there is no potential publication bias in the current meta-analysis.

### Discussion

The results of our meta-analysis, which included 1,926 PD patients and 2,460 healthy controls from 13 countries, demonstrated a high clinical utility of TCS in the diagnosis of PD, with a pooled sensitivity (83%) and specificity (87%). The AUC (0.9306) and DOR (42.89) further indicate an excellent overall accuracy. In addition, a PLR value of 6.94 (95% CI: 5.09–9.48), which is more clinically meaningful for our measures of diagnostic accuracy^38^, suggests that patients with SN hyperechogenicity have a moderate increase in the likelihood of having PD.

For all meta-analyses, heterogeneity is a potential problem when interpreting the results. One major source of heterogeneity is the threshold effect in which different cut-offs are used in the studies included in a meta-analysis. The Spearman correlation coefficient in our study indicates that there is no threshold effect related heterogeneity. Furthermore, meta-regression analysis to find other possible sources of heterogeneity, including geographical location (Europe, Asia or America), sample size (<50 or ≥ 50), age of PD patients (<65 or ≥ 65), ultrasound equipment (<2.5 MHz or ≥ 2.5 MHz), and QUADAS-2 scores (<10 or ≥ 10), revealed that none of the variables were substantial sources of heterogeneity. Therefore, we subsequently performed sensitivity analyses to explore the possibility of significant overall inter-study heterogeneity and to verify the consistency of our results. No obvious alterations were detected, indicating no conceivable source of heterogeneity and statistically consistent results.

In recent years, applications of TCS in the clinical differentiation of PD patients from the healthy population have shown great value. Investigations into the differential diagnosis of PD from atypical parkinsonian syndrome (APS), essential tremor (ET), restless leg syndrome (RLS), or other neurological diseases utilizing TCS suggest that normal SN echogenicity was correlated with multiple system atrophy (MSA)^39^ and ET^4^,^17^,^26^,^27^. Furthermore, SN hypoechogenicity was detected in patients with RLS^21^. More interestingly, abnormal SN hyperechogenic areas were also discovered in 67% of amyotrophic lateral sclerosis (ALS) patients^16^, a disease that might be related to impairment of the nigrostriatal system based on neuroimaging data^40^,^41^. Additionally, lenticular nucleus hyperechogenicity in combination with third-ventricle dilatation of more than 10 mm by TCS helps differentiate progressive supranuclear palsy (PSP) from PD^39^. Moreover, the combination of TCS and olfactory test^42^ or MIBG myocardial scintigraphy^10^ has been identified to improve the differential diagnostic power for identifying PD. All of these investigations demonstrated that the clinical application of TCS may not only help identify PD patients, but also differentiate PD patients from other movement disorders, which suggests great value for TCS in routine clinical practice.

The origin of SN hyperechogenicity, assessed by animal and postmortem studies, has been shown to be related to midbrain iron deposition^43^. Furthermore, the levels of H- and L-ferritins^44^, iron metabolizing protein^45^, plasma ferroxidase activity^46^, and serum CRP^47^ were abnormal in PD patients with SN hyperechogenicity, which further bolsters the concept that SN hyperechogenicity is related to alterations in iron metabolism in PD. Other sources of SN hyperechogenicity include microglia activation^48^ and gliosis^49^, which were found in brain tissue with SN echogenicity after correction for iron and neuromelanin contents. The LRRK2 gene, an autosomal-dominant PD gene, participates in the regulation of neuroinflammation^50^ and microglia activation^51^, and has been found to correlate with SN echogenicity as well. Specifically, carriers of the LRRK2 mutation with no clinical manifestation of PD have a similar proportion of SN hyperechogenicity when compared with idiopathic PD patients^5^. Other PD related gene mutation loci, such as PINK1^52^, GBA^53^ have been also reported to correlate with diverse echogenicity. In the previous research^54^, we explored the potential correlation between SN hyperechogenicity with dopaminergic function represented by DAT-SEPCT, however the results consistent with other study^55^, demonstrated SN echogenicity was not based on dopaminergic pathomechanisms.

Ever since Becker G, *et al.*^3^ first reported a specific high echogenic area within the SN of PD patients over 20 years ago, midbrain echo-features of PD patients have been confirmed and further investigated by numerous groups. However, the utility of TCS in the clinical diagnosis of PD is not universally accepted for several reasons. When a physician wants to utilize a clinical tool, the first parameters examined are the sensitivity and specificity. Unfortunately, different groups report inconsistent results^4^,^5^ due to small sample sizes, and this leads to varied sensitivity and specificity values which precludes the application of TCS for the diagnosis of PD. Therefore, we sought to perform a comprehensive study to evaluate the diagnostic accuracy of TCS. Our study, containing 1,926 PD patients and 2,460 healthy controls from 13 countries, revealed a high pooled sensitivity and specificity, which strongly indicates that TCS could be applied as a clinical tool for the diagnosis of PD patients from healthy controls. Nevertheless, some technical shortcomings must be acknowledged.

One inevitable problem that a sonographer may confront is transcranial insonability. In European populations, 4–15% of participates were found to have an insufficient temporal window^5^,^9^,^16^,^17^,^24^,^25^. However, the value rises to 15–60% in Asian populations^10^,^11^,^21^,^22^,^28^,^34^. This high recording failure rate in TCS application would mostly affect patients of advanced age with female gender^56^ or patients with a small temporal window seen in Asian populations. Recently, high-resolution ultrasound systems with standardized settings or with automated segmentation technique were reported to reduce inter-observer and intra-observer variability^57^, which may help improve TCS image quality and decrease the incidence of insufficient temporal window. Moreover, a novel approach using transcranial B-mode sonography, a 3-D ultrasound platform, was shown to be technically feasible and less dependent on sonographer experience or good bone windows^58^. These innovations and developments in ultrasound systems may effectively improve the application value and diagnostic accuracy of TCS.

To our knowledge, this is the first systematic review and meta-analysis assessing the overall diagnostic accuracy of TCS in PD. A thorough literature search and careful data extraction were performed to avoid any bias. Nevertheless, limitations still exist in our study. First, although we carefully explored the heterogeneity by meta-regression and sensitivity analyses, notable heterogeneity was still observed, which can be due to random variation between individual studies^59^. Second, failure to acquire unpublished data or studies not published in English or Chinese for language limitation may affect the validity of our results.

In conclusion, our systematic review and meta-analysis suggest that TCS has high diagnostic accuracy in the diagnosis of PD patients from the healthy population. As a non-invasive, non-radioactive and convenient neuroimaging technique, application of TCS in routine clinical practice is of great value in the diagnosis of PD. However, large cohorts of high-quality prospective studies are still required to further confirm the value of TCS in the diagnosis of PD.

## Additional Information

**How to cite this article**: Li, D.-H. *et al.* Diagnostic Accuracy of Transcranial Sonography of the Substantia Nigra in Parkinson’s disease: A Systematic Review and Meta-analysis. *Sci. Rep.* **6**, 20863; doi: 10.1038/srep20863 (2016).

## Figures and Tables

**Table 1:** Characteristics of included studies.

Author	Year	Country	PD cases	Age (Ave.)	Diagnostic Criteria	TCS device	Cut-off value	TP	FP	FN	TN	QUADAS score
Stenc Bradvica I	2015	Italy	59	67.2	UK Brain Criteria	2–4 MHz	20 mm^2^	37	6	22	20	11
Maria Sierra	2013	Spain	68	68.93	UK Brain Criteria	2.5 MHz	20 mm^2^	65	7	3	39	10
Sinem Tunc	2015	Germany	53	73.92	UK Brain Criteria	2–2.5 MHz	25 mm^2^	40	21	13	207	10
M. O. Izawa	2011	Japan	33	64.8	UK Brain Criteria	2 MHz	16 mm^2^	26	2	7	30	9
Hee Young Shin	2011	Korea	24	62.3	UK Brain Criteria	2.5 MHz	20 mm^2^	21	4	3	21	11
Tobias Bottcher	2013	Germany	12	60.9	UK Brain Criteria	2.5 MHz	24 mm^2^	10	4	2	28	10
Christoph Schmidauer	2005	Austria	20	64	UK Brain Criteria	2.5 MHz	20 mm^2^	19	5	1	15	10
Pavel Ressner	2007	Czech	47	64.7	UK Brain Criteria	2–3 MHz	19 mm^2^	41	2	6	37	11
Heike Stochner	2007	Austria	100	65.2	UK Brain Criteria	2.5 MHz	24 mm^2^	75	3	25	97	10
Panteha Fathinia	2012	Germany	31	63.5	UK Brain Criteria	3 MHz	20 mm^2^	26	3	5	70	10
Kristina Lauckaitel	2012	Lithuania	71	63.8	UK Brain Criteria	1.3–4 MHz	20 mm^2^	66	8	5	63	11
Edson Bor–Seng–Shu	2014	Brazil	20	62.5	UK Brain Criteria	2–3 MHz	22 mm^2^	20	2	0	7	10
U. Walter	2001	Germany	30	68.9	UK Brain Criteria	2.5 MHz	20 mm^2^	30	7	0	23	10
Philipp Mahlknecht	2013	Austria	17	81.8	UK Brain Criteria	2.5 MHz	18 mm^2^	15	103	2	344	9
Do–Young Kwon	2010	Korea	63	64.6	UK Brain Criteria	2.5 MHz	20 mm^2^	51	5	12	35	11
Yu–Wen Huang	2007	Chinese Taipei	80	59.1	UK Brain Criteria	2.25 MHz	20 mm^2^	54	6	26	114	11
Rita de Cassia	2011	Brazil	17	66.9	UK Brain Criteria	1.6–2.5 MHz	20 mm^2^	15	2	2	9	9
Sabine Mehnert	2010	Germany	183	66	UK Brain Criteria	1.8–3.6 MHz	20 mm^2^	173	8	10	193	10
Nikola Kresojevi	2012	Germany	54	61.5	None	2.5 MHz	19 mm^2^	46	5	8	48	11
Wei–Feng Luo	2011	China	110	58.7	UK Brain Criteria	None	20 mm^2^	88	11	22	99	10
Kristina Lauckaite	2014	Lithuania	141	64.4	UK Brain Criteria	None	20 mm^2^	106	18	35	83	10
Li Chen	2013	China	170	61.3	UK Brain Criteria	1–3 MHz	20 mm^2^	139	12	31	91	9
Sheng Yujing	2011	China	78	62.2	UK Brain Criteria	2.5 MHz	20 mm^2^	66	5	12	55	11
Zhang Yingchun	2010	China	80	60.7	UK Brain Criteria	2–2.5 MHz	20 mm^2^	58	10	22	70	10
Ahmad Chitsaz	2013	Iran	43	63.39	UK Brain Criteria	2–4 MHz	20 mm^2^	39	4	4	46	11
Jurgen Prestel	2006	Germany	42	64.6	UK Brain Criteria	2.5 MHz	20 mm^2^	36	6	6	29	11
Ji Youn Kim	2007	Korea	35	56.7	UK Brain Criteria	2–5 MHz	20 mm^2^	29	2	6	25	10
Jung Ho Ryu	2011	Korea	19	68.5	UK Brain Criteria	2.5 MHz	20 mm^2^	16	24	3	11	10
Wang Rong	2011	China	34	64.11	UK Brain Criteria	1–5 MHz	20 mm^2^	31	4	3	34	9
Araceli	2014	Germany	97	67	UK Brain Criteria	2.5 MHz	21 mm^2^	80	15	17	117	11
Alonso												
Canovas												
W. Ambrosius	2014	Poland	95	62	UK Brain Criteria	2.5–3.5 MHz	19 mm^2^	78	10	17	85	11

Age (Ave.): average of age of included PD Patients; TP: true positive; FP: false positive; FN: false negative; TN: true negative.
